# Recent Discoveries of Novel Mammarenaviruses Infecting Humans and Other Mammals in Asia and Southeast Asia

**DOI:** 10.1080/21505594.2023.2231392

**Published:** 2023-07-02

**Authors:** Brigitte Flannery, Michaela Cain, Hinh Ly

**Affiliations:** Department of Veterinary & Biomedical Sciences, College of Veterinary Medicine, University of Minnesota, Twin Cities, MN, USA

**Keywords:** Mammarenavirus, arenavirus, Wenzhou virus, Plateau pika virus, Lassa virus, Junin virus

## Abstract

Mammarenaviruses, a genus of the family *Arenaviridae*, are capable of infecting mammals and are primarily found in rodent reservoirs worldwide. Mammarenaviruses can be transmitted to humans through contact with infected rodents, and though infection is often asymptomatic, some members of this genus can cause viral haemorrhagic fever which has mortality rates ranging from 1% to 50%. These viruses are typically restricted geographically, based on the geographical range of their host reservoirs. Lymphocytic choriomeningitis virus (LCMV) was previously thought to be the only mammarenavirus found across the globe. However, recent discoveries of two novel human mammarenaviruses, Wenzhou Virus (WENV) and Plateau Pika Virus (PPV), in Asia and Southeast Asia show that mammarenaviruses are more widespread than previously thought. This editorial article aims to raise awareness about these emerging viruses, their genetic and ecological diversities, and clinical significance, and to encourage further study of these emerging viruses.

Mammarenaviruses are enveloped bi-segmented ambisense viruses [1–4], capable of infecting mammals, including humans [5]. They are most commonly transmitted by unintentional human contacts with infected virus-ladened rodent urine, droppings, or nesting materials [1]. Mammarenaviruses are generally divided into two groups, New World and Old World, based on their phylogenetic, serological, and geographical differences [4]. New World (NW) mammarenaviruses, including several known human pathogens, such as Junin, Machupo, Sabia, Chapare, and Guanarito viruses, are found in North and South America in rodent subfamilies *Sigmodontiae* and *Muridae*. Old World (OW) mammarenaviruses are predominantly found in *Murinae* and *Muridae* subfamily rodent reservoirs in Africa and include human pathogens such as lymphocytic choriomeningitis virus (LCMV), Lassa virus (LASV), and Lujo (LUJV) viruses. LCMV is found worldwide due to its natural infection of the common house mouse (*Mus musculus*) [5]. While the rodent reservoir hosts rarely display any symptoms of infection [6], human mammarenaviral infections usually begin with flu-like symptoms and can progress to more severe forms of the disease that can include neurologic disorders, thrombocytopenia, leucopenia, or hearing loss [7]. Importantly, under certain circumstances, several NW and OW mammarenaviruses can cause viral haemorrhagic fevers in humans, which are characterized by vascular leakage, organ failure, shock, and eventual death.

LASV alone can cause up to 300,000 infections per year resulting in 5,000 deaths annually, and one out of every three survivors suffer from often irreversible hearing loss [2]. LASV also has the second highest global burden among all known viral haemorrhagic fevers after dengue fever. It was estimated that about 37.7 million people are currently at risk of contracting LASV in the African continent [2]. Although LASV typically has a mortality rate of 1% in general, the mortality rates can increase to 5–15% in hospitalized patients and 30–50% during outbreaks [8,9]. Currently, there are no approved vaccines against mammarenaviruses, except for the vaccine Candid#1 against Junin virus (JUNV) that is approved for use only in Argentina, where it is endemic [8]. Although antiviral ribavirin is approved for use against mammarenaviral infections, it is only effective if given in the first few days of the infection, which is often not practical due to the asymptomatic nature of the early phase of infection [9].

It was previously thought that LCMV was the only mammarenavirus circulating worldwide. In recent years, however, multiple novel mammarenaviruses have been identified in Asia and Southeast Asia. For example, a recent surveillance study done by Li and colleagues tested 351 rodents for mammarenaviral infections via PCR testing and found 42 positive samples for genetic materials indicative of a mammarenavirus. From those positive samples, a novel mammarenavirus, called Wenzhou Virus (WENV), was isolated from the Wenzhou region of China with sequences of the complete small (S) and large (L) genome segments clustered with other known OW mammarenaviruses on the phylogenetic tree (Figure 1). WENV was found in *Rattus* and *Retiviver* rats as well as in *S. murinus*, which are the Asian house shrews [10].

**Figure 1.**
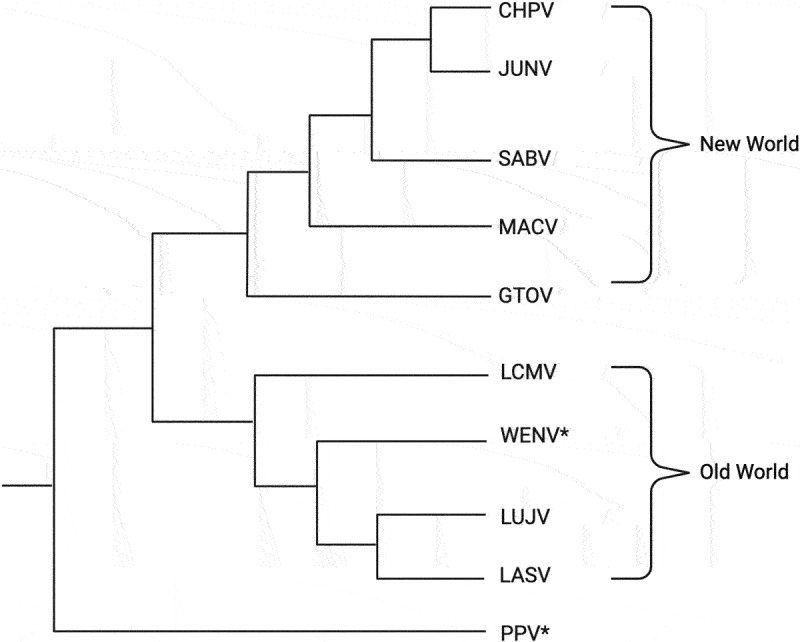
Representative phylogenetic tree indicating relatedness between Old World (OW), New World (NW), and novel mammarenaviruses (WENV and PPV), which are noted with asterisks (*). Phylogenetic tree was built using sequences of the viral large (L) genomic segment of the different mammarenaviruses. Mammarenaviruses included in the phylogenetic tree are Chapare virus (CHPV), Junin virus (JUNV), Sabia virus (SABV), Machupo virus (MACV), Guanarito virus (GTOV), Lymphocytic choriomeningitis virus (LCMV), Wenzhou virus (WENV), Lujo virus (LUJV), Lassa virus (LASV), and Plateau Pika Virus (PPV).

Interestingly, another study revealed similar strains of the OW WENV in Southeast Asian countries, including Cambodia and Thailand, with seropositivity in 89 of 510 (17.4%) samples of patients who tested negative for influenza and dengue but exhibited flu-like symptoms. In the asymptomatic group, a seropositivity rate of 13% was found in 70 of 529 samples from seemingly healthy individuals [3]. The mean age of positive patients was 13 years old, and the highest antibody prevalence was detected in the 6- to 10-year-old age group (40.5%) which suggests the paediatric population is most at risk for infection [3]. Mammarenaviral RNA related to WENV was also identified in multiple rodent species (including *Rattus norvegicus, Bandicota indica*, and *Rattus exulans*) via RT-PCR, with 27 of 627 (4.3%) positive samples from seven locations in Cambodia and Thailand. Similar seropositivity rates of this virus in symptomatic and asymptomatic humans, as well as its detection in geographically disparate rodent species, suggest that WENV has become endemic in these countries.

More recently, another novel mammarenavirus, called Plateau Pika Virus (PPV), has been isolated from plateau pikas, which are small mammals of the *Lagomorpha* family, in the Qinghai Tibet Plateau region [3]. Based on phylogenetic analyses of the genetic sequences of the viral matrix Z protein, the RNA-dependent RNA polymerase (RdRp) L protein, glycoprotein (GP), and nucleoprotein (NP), it is likely that PPV diverged between 77 and 88 million years ago, which would place this newly identified mammarenavirus in a separate lineage that appeared long before the divergence of OW and NW mammarenaviruses (Figure 1) [4]. RNA sequencing of plateau pika intestinal contents revealed PPV RNA in 34 of 541 (6.3%) of the samples analysed. Serological screening of human sera detected antibodies in 8 of 335 (2.4%) samples [4]. PPV was shown to infect and replicate in multiple mammalian cell lines, including human, primate, and rodent cells, suggesting that the host range of PPV could spread beyond rodents and small mammals if exposed to new hosts. It is important to note that PPV can cause haemorrhage in the spinal cord, inflammatory infiltrates, oedema in the cerebral cortex, and central nervous system in immunocompromised mice, suggesting its potential pathogenicity in other immunocompromised hosts, including potentially humans [4].

Recent discoveries of novel mammarenaviruses in wider mammalian species, in combination with seropositivity in humans, suggest that the host range for some mammarenavirus infections is wider than previously thought and may be expanding. The wide genetic variations of pathogenic mammarenaviruses could result in increased probability of spillover events with human exposure to many different mammals that can carry these mammarenaviruses in the wild. This increased prevalence of mammarenaviruses in human populations, spillover potential, and their potential to cause severe disease calls for more attention paid to the epidemiological, ecological, virologic, immunological, and pathogenesis investigations into these emerging viral pathogens. These studies will inform the development of prophylactic and therapeutic treatments to protect humans against these novel viruses with pathogenic and pandemic potentials.

## Data Availability

No primary data is included in this article.
